# Periostin Induces Epithelial‐Mesenchymal Transition via p38‐MAPK Pathway in Human Renal Tubular Cells by High Glucose

**DOI:** 10.1002/iid3.70077

**Published:** 2024-11-21

**Authors:** Xiaoling Xiong, Xing Feng, Yuqing Ding

**Affiliations:** ^1^ Department of Nephrology, Sir Run Run Shaw Hospital, College of Medicine Zhejiang University Hangzhou China; ^2^ Department of Thoracic Surgery, Affiliated Hangzhou First People's Hospital, School of Medicine Westlake University Hangzhou China

**Keywords:** high glucose, HK‐2 cells, p38‐MAPK signaling, periostin

## Abstract

**Background:**

Periostin mediates inflammation and fibrosis by regulating extracellular matrix adhesion, migration, and differentiation in multiple organ diseases. Studies have shown periostin mainly located in the dilated mesangium, tubulointerstitial and fibrotic regions of the diabetic kidney disease, which was negatively correlated with renal function. However, the underlying mechanism remains poorly explored.

**Methods:**

The expression of periostin in HK‐2 cells was investigated under high glucose and high concentration of TGF‐β1. The signaling pathway of periostin involved in epithelial‐mesenchymal transdifferentiation of HK‐2 cells was also validated. The expression of periostin were investigated by RT‐PCR, western blot analysis and immunofluorescence assays with different concentrations of glucose and TGF‐β1. The expression of E‐Cad, α‐SMA and p38 proteins were also detected. The effects of periostin, E‐Cad, and α‐SMA in high glucose were investigated by p38 inhibitors. To demonstrate the interaction among periostin, p38 and EMT markers, periostin under high glucose and high TGF‐β1 was knocked down, resulting p38 and phosphorylated p38 was evaluated.

**Results:**

The combined of high glucose (HG, 22 mmol/L) and high TGF‐β1 (10 ng/mL) upregulated the expression of periostin obviously, stimulating the expression of α‐SMA and p38 while inhibiting the expression of E‐Cad. p38 inhibitors reduced the expression of periostin and α‐SMA while promoted E‐Cad protein expression in HK‐2 cells under HG conditions. Additionally, p38‐MAPK signal pathway was involved in epithelial‐mesenchymal transition of human renal tubules in high glucose environment. Significant, knockdown periostin expression effectively inhibited the expression of p38 and phosphorylated p38 under the combination of HG and high TGF‐β1, verifying the interaction of periostin with the p38‐MAPK signaling pathway.

**Conclusion:**

Periostin, a downstream factor of TGF‐β1, is positively regulated by TGF‐β1 under HG condition, affecting the epithelial‐interstitial differentiation of HK‐2 cells via p38‐MAPK signaling pathway. Therefore, periostin may serve as a biomarker of renal fibrosis in diabetic kidney disease.

## Graphical abstract

Periostin, a downstream factor of TGF‐β1, is positively regulated by TGF‐β1 under high glucose condition, affecting the epithelial‐interstitial differentiation of HK‐2 cells via the p38‐MAPK signaling pathway. Therefore, periostin may serve as a biomarker of renal fibrosis in diabetic kidney disease.

## 1 Introduction

Diabetic kidney disease (DKD) is the leading cause of end‐stage renal disease in patients with diabetes mellitus. Its histological changes mainly affect the glomerular filtration units, including the thickening of the basement membrane, mesangial cell proliferation, endothelial cell change and podocyte injury. The pathogenesis of DKD is complex and relatively related to metabolic disorders, hemodynamic changes, inflammation and fibrotic factors, which ultimately promotes the progression of DKD in patients with type 2 diabetes. Tubulointerstitial lesions occurring in glomerular and renal angiopathy is closely related to the prognosis of diabetes. However, there are no new prognostic biomarkers for conventional clinical analysis or ongoing clinical trials currently except estimated glomerular filtration rate (eGFR) and proteinuria [1, 2]. Klessens et al. [3] found the prevalence of 3 DKD deaths was higher than clinical diagnosis via an autopsy study. 20% of diabetic patients showed typical histopathological changes of DKD without any proteinuria or eGFR below 60 mL/min. These studies manifest the unpredictable progression of DKD which becomes an urgent for developing new strategies to better assess disease progression. Although some candidate biomarkers have been described in previous studies, only a few may contribute to identifying the progressive stage of DKD.

Renal interstitial fibrosis (RIF) is a common pathway and pathological change in all chronic kidney diseases with progression to chronic kidney failure. The key mechanism driven RIF is epithelial‐mesenchymal transition (EMT), which activates tubular epithelial cells (such as HK‐2 cells) into myofibroblast (Myofibroblasts, MyoF), contributing the secretion of collagen deposited in renal interstitium and initiating fibrosis [3, 4, 5, 6]. Calcium‐dependent transmembrane glycoprotein (E‐cadherin, E‐cad) showed a decrease number of HK‐2 cells represented by E‐cad, while the increase of interstitial cells marked by α‐smooth muscle actin (α‐SMA).

Periostin, known as osteoblast‐specific factor 2 (OSF‐2), is a member of the extracellular matrix protein family. Periostin contains four distinct domains derived from homologous insect domains associated with neuronal adhesion. Periostin protein mediates signaling in the extracellular and external environment through interaction with proteins such as collagen‐1, Notch‐1, BMP‐1, and various cell surface integrins in the extracellular matrix (ECM) [2], regulating ECM adhesion, migration and differentiation. Substantial studies have shown that periostin plays a role in mediating inflammation and fibrosis in a variety of organs (e.g., heart, lung, skin, liver, skeletal muscle and retina) [6, 7, 8, 9, 10, 11, 12].

Periostin, initially found in epithelial cells and cystic fluid of the autosomal dominant polycystic kidney, could accelerate the proliferation of cystic wall epithelial cells and promote the remodeling of renal interstitial in renal disease [13]. In the renal tissues of patients with focal segmental glomerulosclerosis, IgA nephropathy, and lupus nephritis, periostin expression were significantly upregulated. Periostin protein is mainly distributed in the dilated mesangium, renal tubulointerstitium and fibrotic areas, and its expression level was negatively correlated with renal function [14]. Related studies [15] have found that urinary periostin which was closely associated with the progression of IgA nephropathy could predict renal failure. In a study of kidney transplant recipients, the level of urinary periostin could effectively distinguish recipients between chronic transplant kidney and normal ones [16]. Cho et al. [17] induced the progression of diabetes in Periostin‐null mice and wild‐type mice with chain‐zotocin administration after unilateral nephrectomy. and found that periostin deficiency attenuated kidney fibrosis in diabetic nephropathy by improving pancreatic β‐cell dysfunction and reducing kidney EMT. Bian et al. [18] demonstrated that reducing periostin alleviated the activation, fibrosis, and inflammation of the 5/6 nephrectomy‐induced intrarenal renin‐angiotensin system in rats. However, the signal pathway between periostin and EMT remains poorly elucidated in DKD.

In our work, the expression of periostin in HK‐2 cells under high glucose and high concentration of TGF‐β1 was evaluated. To determine the signal pathway, periostin in epithelial‐mesenchymal transdifferentiation of HK‐2 cells was subsequently investigated. The results indicated periostin could participated in the development of RIF in DKD which might serve as a potential biomarker of renal disease progression.

## 2 Materials and Methods

### 2.1 Materials

Glucose (50‐99‐7) and p38 inhibitor (Hy‐10256) were purchased from Bio‐Rad. Periostin antibodies (DF6746), α‐SMA antibody (AF1032) were obtained from Affinity. E‐Cad(3195), p‐p38 (thr180/tyr182) antibody (4511) and p38 antibody (9212) were purchased from Cell Signaling Technology (CST). IP cell lysate (P0013B), PMSF (P105539), BCA protein concentration detection kit (P0010) were acquired from Biotech. Trizol (Lot:252250AX), TB Green Premix Ex TaqII kit (RR420Q/A/B), PreimerScript RT reagent Kit (RR037A) and other biochemical reagents were purchased from Beyotime Biotech. Goat anti‐rabbit IgG Secondary Antibody (Alexa Fluor 488) (A11008) and Lipofectamine 3000 Reagent（L3000001）was purchased from Thermal Fisher Scientific Company. 4′, 6‐diamino‐2‐benzene (DAPI) (D9542) from Sigma, TGF‐β1 (P01137) was purchased from Kingsley. Fetal bovine serum (FBS) (FSD500), DMEM medium (PM150210) were purchased from Wuhan YiBiotechnology Co., Ltd. Related consumables (Petri dishes, Tip heads, EP tubes, etc.) were purchased from Thermal Scientific Fisher Company.

### 2.2 HK‐2 Cell Culture

All human renal tubular epithelial cell line (HK‐2) was obtained from the American Type Culture Collection Corporation (ATCC, Shanghai). DMEM medium containing 10% FBS, 37°C, 5% CO_2_ and saturated humidity were maintained for culture. The 3–5 passage cells were tested in vitro. The third passage cells were observed and used for further experiments.

HK‐2 cells were cultured in DMEM medium with different concentration of TGF‐β1 (0.1, 1 and 10 ng/mL) and different concentration of glucose (5.5, 11, 22 mmol/L), respectively. Normal glucose (recorded as CK) was set as blank group. The cells were carried out for subsequent experiments before being cocultured for 3 days. Then high glucose (HG), TGF‐β1 and HG + TGF‐β1 groups were established for co‐incubated with HK‐2 cells.

To determine the influence of p38 inhibitors (p38 i) to HK‐2 cells under HG, cells were cultured with 60 nM p38 i under HG condition. The fluorescence of periostin was detected by an inverted fluorescent microscope (IX73, OLYMPUS). The expressions of E‐Cad, α‐SMA and periostin were conducted via Western Blot. CK was set as blank group while HG was served as control group during the experiments.

### 2.3 Cell Transfection Experiments

HK‐2 cell periostin high‐expressed model was established under HG (22 mmol/L) and TGF‐β (10 ng/mL). Then, the model was treated with periostin siRNA (labeled P i). The groups were list as follows: CK, HG + TGF‐β1, HG + TGF‐β1 + P i. The siRNA was synthesized by Shanghai Biotech with the following sequence in Table 1.

**Table 1:** The sequence of siRNA.

Name	Sequences
hsa‐periostin siRNA‐1203	GGAACGUUAUGAAGCAUCATT
	UGAUGCUUCAUAACGUUCCTT
NC	UUCUCCGAACGUGUCACGUTT
	ACGUGACACGUUCGGAGAATT

SiRNA (0.67 μg, 50 pmol) was added to a certain amount of serum free dilution and fully mixed. Lipofectamine 3000 was extracted and added to the above solution with a final volume of 25 μL maintaining for 5 min at room temperature to form a complex. Then the above solution was added to 450u1 medium containing HK‐2 cells incubating for 6 h, change the medium and continued for 2 days for the subsequent assays.

### 2.4 Real‐Time Quantitative PCR (RT‐PCR)

Cell samples were collected, lysed with 1 mL Trizol to extract total RNA. Then, cDNA was generated at 37°C for 60 min, followed by 85°C for 5 min to terminate the reaction. Next, PCR amplification was performed using the TB Green Premix Ex TaqⅡ kit. Amplification parameters were set at 95.0°C initialization for 15 min, then 95.0°C heating for 10 s, 59.0°C annealing for 20 s, 72.0°C extension for 30 s, and the above steps were repeated 40 cycles. Melt Curve from 65 to 95°C. Increment 0.5°C for 10 s Plate Read. The OD photometer (high precision) was measured by Merinton SMA4000 and the quantitative PCR was tested by CFX Connect Real‐Time PCR System(BIO‐RAD CFX Manager, America), GAPDH was used as internal reference gene. All primers were purchased from Shanghai Sonny Biotechnology Co., Ltd. The sequence of primers were list as Table 2.

**Table 2:** The sequence of primers.

Name	Sequences
Periostin forward	CTGGCACCTGTGAATA
periostin reverse	CTCCCTTGCTTACTCC
p38 forward	GCCAAGCCATGAGGCAAGAAACTAT
p38 reverse	TCCAATACAAGCATCTTCTCCAGCA
GAPDH forward	AAGGTCGGTGTGAACGGATT
GAPDH reverse	TGAGTGGAGTCATACTGGAACAT

### 2.5 Western Blot

The samples from each group were collected into EP tubes and 200 μL Western and IP lysate were added to fully lysis for 30 min. Subsequently, the above samples were centrifuged at 12,000 rpm in a high speed refrigerated centrifuge (H1650, Shanghai Luxiangyi Co., Ltd.) and the supernatant was removed to obtain total protein. Polyacrylamide gel electrophoresis was prepared for PVDF transmembranes and blocked with 5% nonfat milk powder for 1 h at room temperature. The blocked PVDF membrane was removed and the primary antibodies against α‐SMA (1:1000), E‐Cad (1:1000), Periostin (1:1000), p38 (1:1000), p‐p38 (1:1000) and GAPDH (1:1000) was added and incubated overnight at 4°C before washed with TBST for three times. Goat anti‐Mouse IgG (1:5000, GAM007, Multi Sciences (LianKe)) and Goat anti‐Rabbit IgG (1:5000, GAR0072, Multi Sciences (LianKe)) were added and incubated for 1 h at room temperature. Finally, the ECL chemiluminescent was performed by a transilluminator(ChemiDoc XRS+ System, Bio‐RAD) and quantified by Image J system.

### 2.6 Immunofluorescence Assay

For immunofluorescence, cells were washed by PBS and fixed with 4% paraformaldehyde for 20 min at room temperature. 0.1% Triton X‐100 was then added to break membrane at room temperature for 20 min. Blocking with 5% BSA or 1% FBS for 30 min and washing PBS. Followed by the addition of periostin primary antibody (1:1000) under 4°C overnight respectively. Alexa Flour secondary antibody was added (1:500) and incubated at room temperature for 1–2 h. 10 ng/ml DAPI (1:100 ~ 1:500) was added and incubated at 4°C for 15 min. Finally, sections were occluded and observed by a microscope (ECLIPSE Ti‐S, Nikon, Japan).

### 2.7 Statistical Analysis

All statistical analyses were constructed by Graphpad Prism (version 8.0; Graphpad Software Company). Results were derived from three independent experiments and the measured data were presented as mean ± standard deviation (SD). For the comparison of multiple sets of data, the one‐way analysis of variance (ANOVA) method was used for statistical analysis. *p* ≤ 0.05 (*p ≤ 0.05, **p ≤ 0.01) was considers as significant differences.

## 3 Results

### 3.1 Periostin Expression in HK‐2 Cells Under High Glucose and TGF‐β1 Concentration

**Figure 1:**
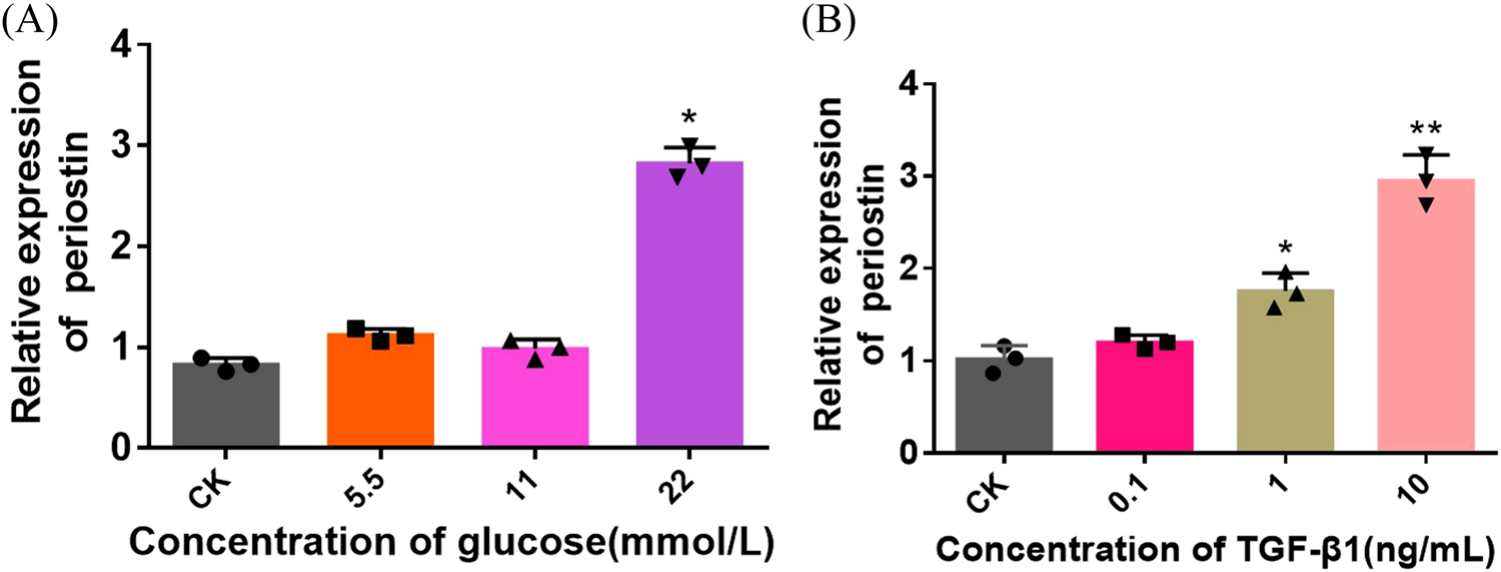
Periostin expression in HK‐2 cells induced by HG and high concentration of TGF‐β1. (A) RT‐PCR was used to detect the effect of different concentrations of glucose on the expression of periostin mRNA. (B) RT‐PCR was used to detect the effect of TGF‐β1 on the expression of periostin mRNA. *p < 0.05; **p < 0.01; * versus CK. CK, blank control group; HG, high glucose; TGF‐β1, transforming growth factor β1.

HK‐2 cells were cocultured with different concentrations of glucose and TGF‐β1 to detect the level of periostin. We compared the expression of periostin in the medium supplemented with different glucose concentrations (5.5, 11, 22 mmol/L). The level of periostin was globally positively correlated with glucose concentration. Compared with blank group, periostin was significantly upregulated at a glucose concentration of 22 mmol/L (Figure 1A). The level of periostin was upregulated with the increase concentration of TGF‐β1. Periostin expression doubled as the concentration of TGF‐β1 reached 10 ng/mL (Figure 1B). Herein, TGF‐β1 at 10 ng/mL and glucose at 22 mmol/L were selected as high glucose and high TGF‐ β1 conditions to evaluate the epithelial‐mesenchymal transdifferentiation of HK‐2 cells.

### 3.2 The Combined Effect of High Glucose and High Concentration of TGF‐β1 on Periostin Expression of HK‐2 Cells

**Figure 2:**
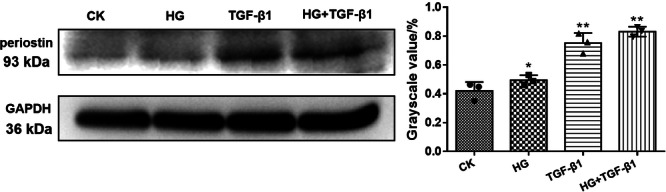
Western blot analysis of periostin protein expression in HK‐2 cells stimulated by HG (22 mmol/L) and high concentration of TGF‐β1 (10 ng/mL); Image J software was used to collect the gray value of protein bands. *p < 0.05; **p < 0.01; * versus CK. CK, blank control group; HG, high glucose; TGF‐β1, transforming growth factor β1.

The expression of periostin protein was estimated when stimulated with HG and high concentration of TGF‐ β1 in HK‐2 cells (Figure 2). The results showed that periostin protein levels under HG, TGF‐β1 and HG + TGF‐β1 were higher than those of CK. Notably, the HG + TGF‐β1 group exhibited the highest protein expression levels, suggesting the combined of HG and High concentrations of TGF‐β1 might synergistically promote the expression of periostin.

### 3.3 HG and TGF‐β1 Affected the Level of Periostin Protein

**Figure 3:**
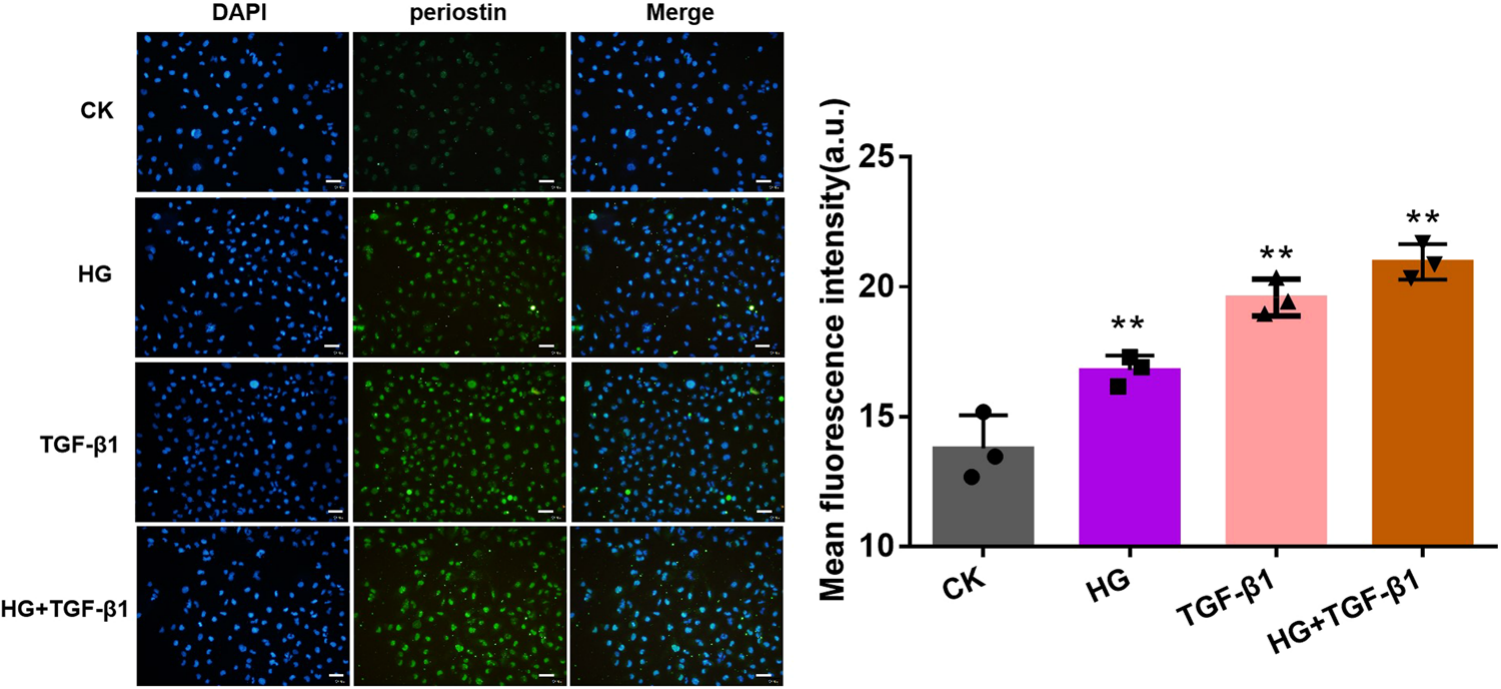
Periostin protein expression in HK‐2 cells cocultured with HG (22 mmol/L), high concentration of TGF‐β1 (10 ng/mL) and HG + TGF‐β1 (22 mmol/L, 10 ng/mL) was observed by immunofluorescence and mean fluorescence intensity of periostin. **p < 0.01; * versus CK. Scale bar: 50 μm. Green: Alexa Fluor 488 labeled periostin; blue: DAPI labeled nucleus. CK, blank control group; HG, high glucose; TGF‐β1, transforming growth factor β1.

The immunofluorescence was observed to co‐localize the periostin and nucleus of HK‐2 cells when cocultured with HG, high concentration of TGF‐β1 and HG + TGF‐β1 (Figure 3). The periostin was labeled by Alexa Fluor 488. Compared with CK group, the treating of HG, TGF‐β1 and HG + TGF‐β1 could promote the expression of periostin in nucleus. Evidently, the combined group attained the strongest fluorescence intensity.

### 3.4 The Effects on E‐Cad, α‐SMA and p38 Protein of EMT Under HG and TGF‐β1 Condition

**Figure 4:**
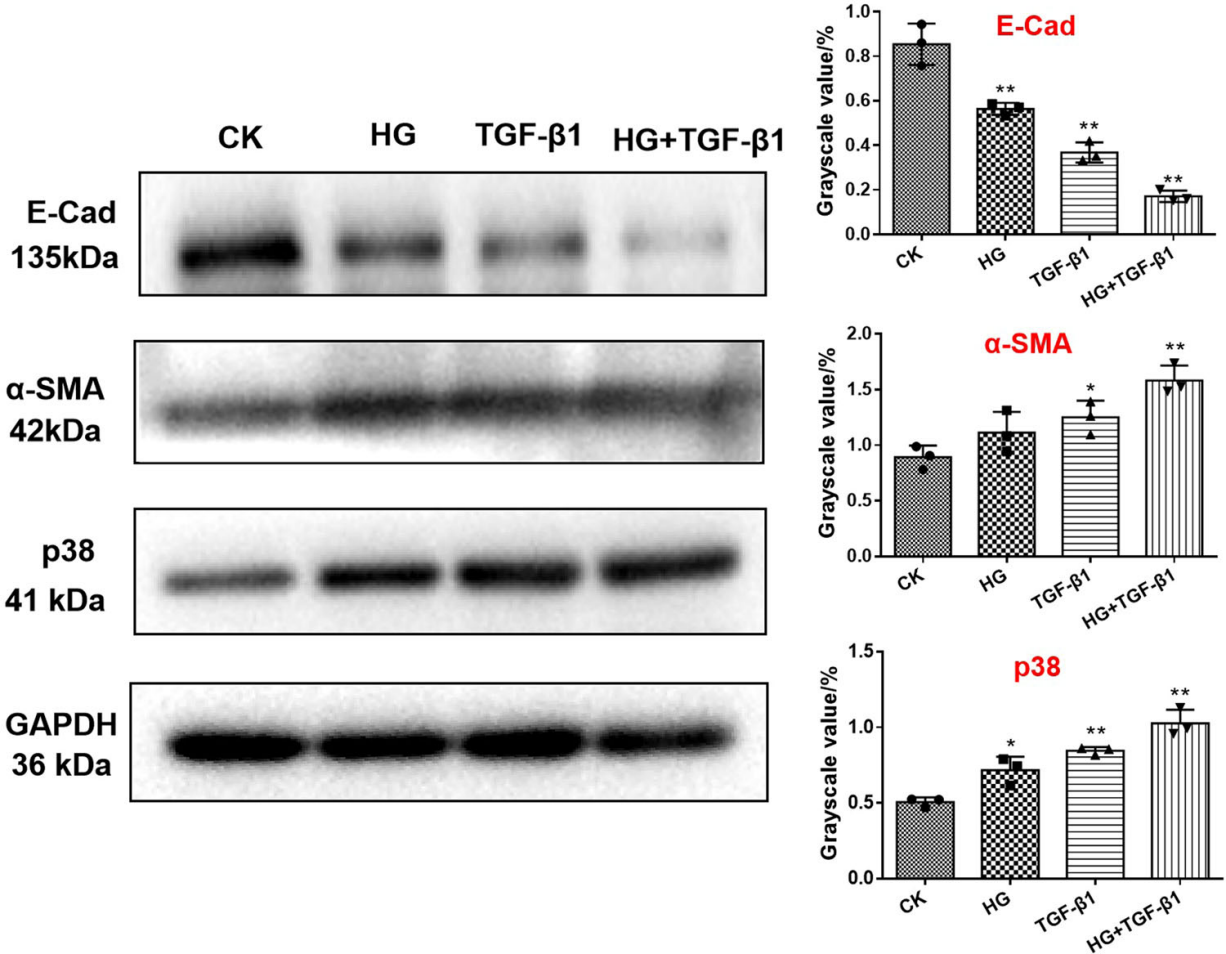
Western blot was used to detect the expression of E‐Cad, α‐SMA and p38 in HK‐2 cells induced by HG (22 mmol/L), TGF‐β1 (10 ng/mL) and HG + TGF‐β1 (22 mmol/L, 10 ng/mL). (D–F) Image J software was used to collect the gray value of protein bands. *p < 0.05; **p < 0.01; * versus CK. CK, blank control group; HG, high glucose; TGF‐β1, transforming growth factor β1.

Western blot was investigated to determine the effects of HG and TGF‐β1 on the signaling proteins associated to the EMT pathway (E‐Cad, α‐SMA and p38). The expression of p38 and α‐SMA proteins induced by HG and High concentration of TGF‐β1 was higher than that of CK group, while the expression level of E‐Cad protein was decreased (Figure 4). Intriguingly, E‐Cad was significantly downregulated, while α ‐SMA and p38 expressed the highest expression in HG + TGF‐ β1 group, which might be the cause of renal fibrosis.

### 3.5 P38 Inhibitor Reversed Periostin Expression and Transdifferentiation of HK‐2 Cells Under HG Condition

**Figure 5:**
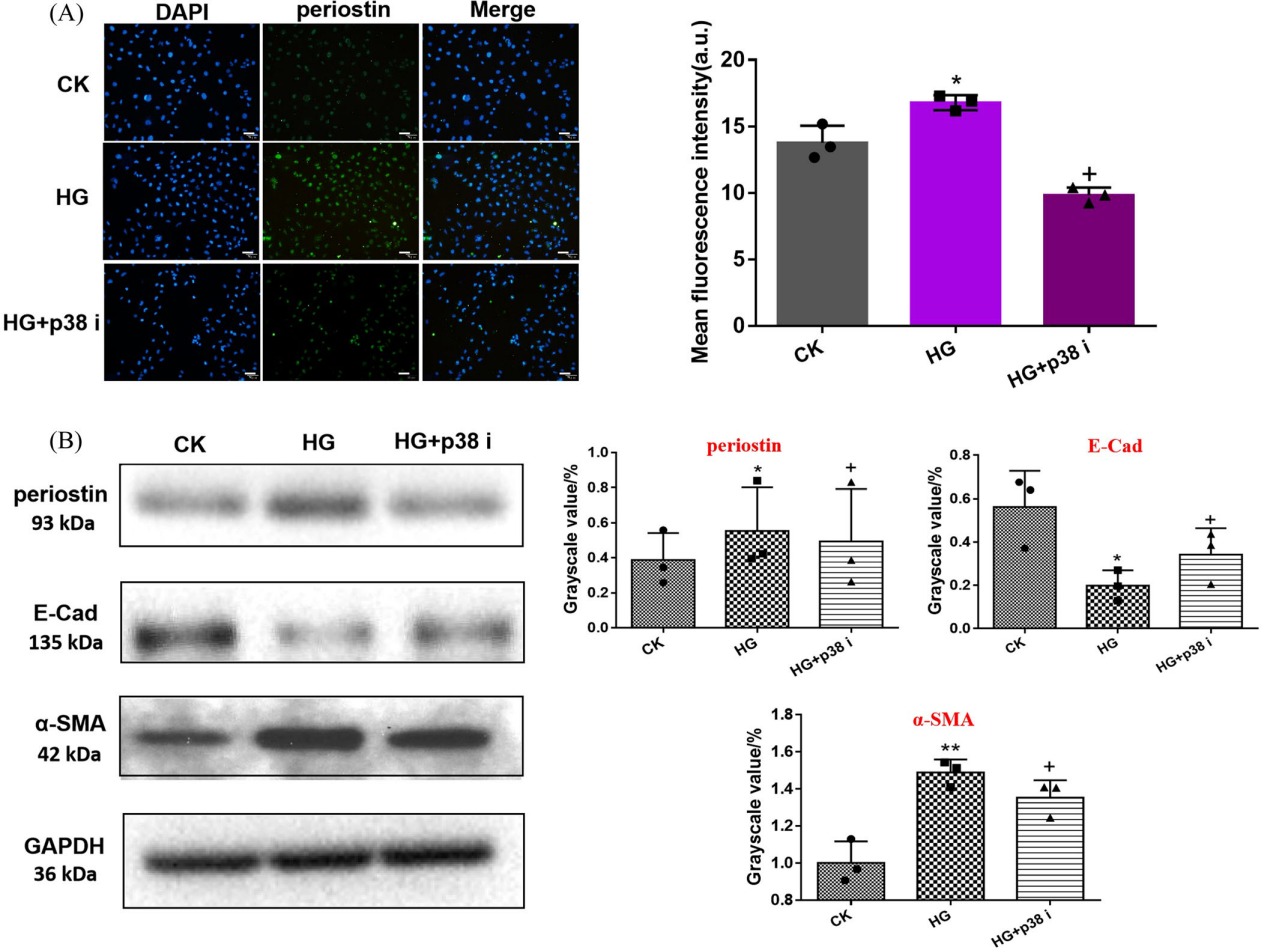
(A) The periostin protein expression in HK‐2 cells stimulated by HG, HG + p38 inhibitor was observed by immunofluorescence and fluorescence intensity. Green: Alexa Fluor 488 labeled periostin; blue: DAPI labeled nucleus. Scale bar: 50 μm. (B) Western blot was used to observe periostin expression in HK‐2 cells stimulated by CK, HG (22 mmol/L), HG + p38 i (22 mmol/L, 60 nmol/L). Image J software was used to collect the gray value of protein bands. *p < 0.05; **p < 0.01; ^+^ *p* < 0.05; * versus CK; ^+^ versus HG. CK, blank control group; HG, high glucose; TGF‐β1, transforming growth factor β1.

As shown in Figure 5A, the immunofluorescence of periostin under CK, HG, and HG + p38 i were observed. The green fluorescence of HG group appeared the strongest. In contrast, HG + p38 i group showed significantly decreased green fluorescence. The level of periostin protein and EMT protein as E‐Cad, α‐SMA under different conditions were detected. The results showed that the expression levels of α‐SMA and periostin in HG and HG+p38 i group were higher than those in CK Group, while the expression level of E‐Cad was significantly downregulated. These results indicated that p38 i could reverse periostin expression and transdifferentiation of HK‐2 cells under HG environment.

### 3.6 Inhibition of Periostin and the Effect of p38‐MAPK Signaling Pathway

**Figure 6:**
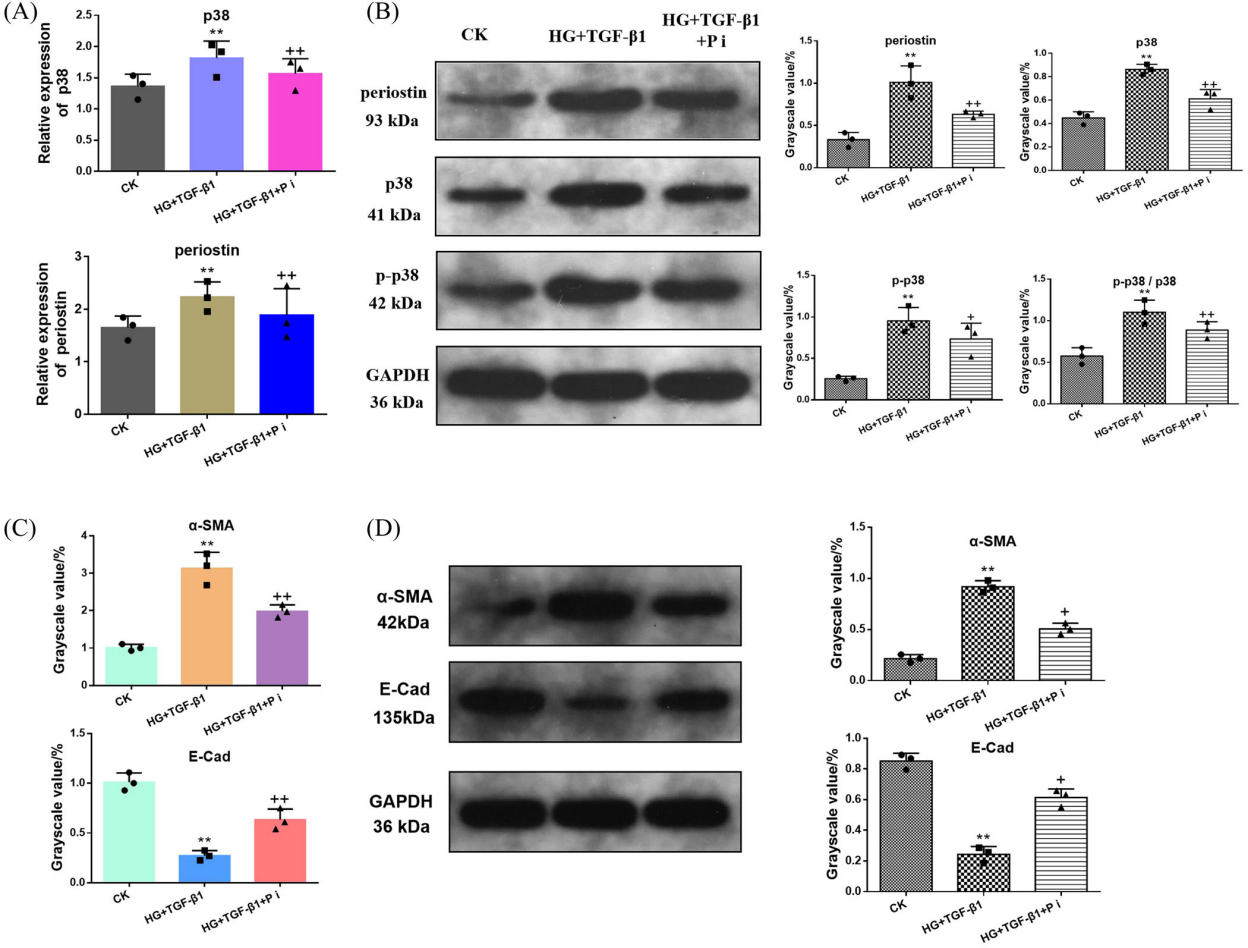
(A) RT‐PCR was used to observe gene expression in HK‐2 cells stimulated by CK, HG (22 mmol/L) + TGF‐β1 (10 ng/mL) and HG + TGF‐β1 + P i (20 μmol/L), respectively. (B) The protein expression in HK‐2 cells stimulated by three different group. Image J software was used to collect the gray value of protein bands. (C) The mRNA expression of α‐SMA and E‐Cad. (D) The protein expression of α‐SMA and E‐Cad, and gray value of countparts detected by Image J. *p < 0.05; **p < 0.01; ^+^ *p* < 0.05; ^++^ *p* < 0.01; * versus CK; ^+^ versus HG + TGF‐β1. P i, periostin inhibitors (siRNA). CK, blank control group; HG, high glucose; TGF‐β1, transforming growth factor β1.

To further clarify the relationship of periostin and p38‐MAPK signaling pathway, HK‐2 cells were co‐incubate with HG and high concentration of TGF‐β1 before siRNA transfected to down‐regulate the expression of periostin, followed by the detection of p38 and its phosphorylation product (Figure 6). The level of p38 gene and phosphorylated p38 (p‐p38) and p38 protein were measured to assess the effect of periostin on the p38‐MAPK signaling pathway. As shown in Figure 6A, the expression of periostin and p38 genes in HG + TGF‐β1 group was upregulated. Distinctly, periostin was reversed after treating with P i (HG + TGF‐β1 + P i group). Additionally, p38 gene also showed the similar trend. Ulteriorly, the expression of p38 and p‐p38 protein and the ratio of p38/p‐p38 was assessed (Figure 6B). The results suggested that knockdown periostin could inhibit the p38‐MAPK signaling pathway. Figure 6C,D revealed the expression of α‐SMA and E‐Cad mRNA and protein under the condition of CK, HG + TGF‐β1 and HG + TGF‐β1 + P i, which indicated that the downregulated periostin was conducive to reverse the EMT process.

## 4 Discussion

Periostin is rarely expressed in healthy tissues, but overexpressed in different renal lesions both in animal and human renal diseases, which is associated with the decreased renal function. Previous studies have discovered periostin was significantly up‐regulate in type 2 diabetes before the onset of significant albuminuria, which was closely associated with DKD progression [14, 19, 20]. However, its potential mechanisms still deserve further study. High concentrations of glucose is the initiator of DKD. It may activate multiple mitogen‐activated MAPK pathways at p38 and P42/44 in a short time by inducing ROS production, causing the increase of TGF‐β1 and ECM in mesangial cells. TGF‐β1, as a profibrotic factor and growth factor, plays an important role in the progression of DKD [21, 22, 23]. TGF‐β1 participates in the whole process of DKD through various signal transduction pathways. Recently, the role of RIF pathogenesis in DKD has gradually attracted much attention.

Researchers have demonstrated that mouse distal collecting duct cells were cultured in vitro and transfected with periostin, cDNA exhibited higher periostin expression during the induction of fibrosis [24]. The deletion of the periostin gene resulted in a significant reduction of pro‐inflammatory mediators, preserving renal structure and function in an animal model of nephrotoxic serum (NTS)‐induced glomerulonephritis [25]. Periostin combined with DNA aptamer successfully inhibited tubulointerstitial fibrosis induced high expression of periostin, and the effects of glucose at 22 mmol/L and TGF‐β1 at 10 ng/mL were significant. This suggested periostin was associated with renal interstitial cell fibrosis and the development of DKD. Um et al. [26] evaluated the effect of periostin inhibition by an aptamer‐based inhibitor on renal fibrosis under diabetic conditions, and found TGF‐β1 treatment significantly upregulated periostin, fibronectin, and type I collagen mRNA and protein expressions in inner medullary collecting duct cells in vitro. Besides, Satirapoj B et al. [27] confirmed that urinary periostin was associated with renal derangement which might be a potential marker of diabetic renal injury. Over activation of TGF‐β was considered to be a key pathogenic factor of diabetes nephropathy. Studies have shown that the miR‐200b/c content in mouse glomerular mesangial cells was significantly upregulated, leading to hypertrophy of glomerular mesangial cells under high glucose or TGF‐β1 stimulation [17, 18]. Spontaneously, our study demonstrated periostin was significantly increased in HK‐2 cells when stimulated with high glucose and high concentration of TGF‐β1, which had a synergistic effect. Periostin, as a downstream factor of TGF‐β1, was positively regulated by TGF‐β1 in high glucose environment.

Reduction of E‐cad and increase of α ‐SMA are hallmarks of transdifferentiation of HK‐2 cells [4]. In our work, periostin expression in HG and high concentration TGF‐β1 environment was negatively correlated with E‐cad and positively correlated with α‐SMA, which illustrated a direct correlation between periostin overexpression and the development of RIF. Subsequently, p38 was stimulated, resulting the same trend as periostin. Combined stimulation (HG + TGF‐β1) to HK‐2 cells effectively promoted the expression of α‐SMA and p38 while inhibited the expression of E‐Cad. The above results strongly confirmed the collective correlation between periostin and EMT and p38 factors.

Previous studies have shown that periostin promoted renal fibrosis through the p38 MAPK pathway after acute kidney injury triggered by hypoxia or ischemia‐reperfusion injury [17]. In HK‐2 cells treated by p38 inhibitor with high glucose stimulus, periostin and α‐SMA protein was decreased while the expression of E‐Cad increased. The results indicated p38 inhibitor had a reverse effect on periostin and EMT in HK‐2 cells under high glucose. To further confirm the role of periostin in RIF, siRNA was transfected to down‐regulate the level of periostin in HG + TGF‐β1 induced HK‐2 cells. The expression of p38 gene and phosphorylated p38 (p‐p38) and p38 proteins verified the role of periostin on the p38‐MAPK signaling pathway, which illustrated a bidirectional regulation of periostin with p38. Our results suggested periostin engaged in activating and presenting signaling factors during the development of renal interstitial transforming lesions. All above confirmed that periostin might be involved in human renal tubular EMT in a high glucose environment by acting on the p38‐MAPK signaling pathway.

## 5 Conclusion

In summary, Our study established a HK‐2 cell model under conditions of HG and high concentrations of TGF‐β1 by inducing renal interstitial cell fibrosis to elucidate the relationship between periostin and renal interstitial transdifferentiation and p38‐MAPK signaling pathway. P38 i could reverse periostin protein expression and transdifferentiation of HK‐2 cells under HG environment. Down‐regulate the periostin gene under HG and high concentration of TGF‐β1, p38 and p‐p38 expression of the p38‐MAPK pathway would also be inhibited. Periostin which could promote epithelial‐mesenchymal differentiation was positively regulated by TGF‐β1 via p38‐MAPK signal pathway under HG environment. However, Due to the complexity mechanism of DKD, periostin may be involved in renal interstitial transformation via other pathways besides p38‐MAPK. This study did not comprehensively evaluate the interaction relationship between periostin and p38‐MAPK pathway, and whether other pathways and mechanisms are involved is still unknown. We aspire to establish clinical samples and in vivo models to verify the potential mechanism of periostin in the development of DKD. Anyway, periostin may become an early diagnostic and accurate evaluation candidate biomarker of RIF in DKD in the future.

## Author Contributions

All authors made a meaningful contribution to the work. **Xiaoling Xiong:** conceptualization, subject design, editing, original draft (lead) and editing. **Xing Feng** and **Yuqing Ding:** experiment, formal analysis, mapping and reviewing.

## Conflicts of Interest

The authors declare no conflicts of interest.

## Data Availability

The experimental data can be reasonably obtained from the corresponding author.
